# Human-Biomonitoring – ein wichtiger Baustein zur Umsetzung der EU-Chemikalienstrategie. Die Arbeit der Human-Biomonitoring-Kommission am Umweltbundesamt

**DOI:** 10.1007/s00103-022-03579-y

**Published:** 2022-09-01

**Authors:** Claudia Röhl, Marike Kolossa-Gehring

**Affiliations:** 1 Umweltmedizin und Toxikologie, Landesamt für soziale Dienste Schleswig-Holstein, Gartenstr. 24, 24534 Neumünster, Deutschland; 2 Toxikologie, gesundheitsbezogene Umweltbeobachtung, Umweltbundesamt, Corrensplatz 1, 14195 Berlin, Deutschland

In unserer modernen Lebenswelt sind chemische Stoffe ein fester Bestandteil des Alltags. Mit jedem Atemzug, jedem Umweltkontakt unserer Haut, mit der Nahrung und dem Trinkwasser nehmen wir sie fortwährend in unseren Körper auf und geben sie zum Teil schon an unsere ungeborenen oder gestillten Kinder weiter. Dies gibt ohne Zweifel Anlass zur Besorgnis, ist aber in der Sache erst einmal nicht unerwartet, denn unser Körper ist ein offenes System, das von und mit Stoffen aus unserer Umwelt lebt. Von den Stoffeigenschaften hängt ab, ob und wie sie sich im Körper und in den Organen verteilen, wie lange sie dort verweilen, wie die Substanzen mit dem Organismus wechselwirken, ob sie verändert und abgebaut werden und ob und wie sie wieder ausgeschieden werden.

Die Gesamtheit dieser Teilprozesse, die in komplexen biologischen Systemen wie dem menschlichen Körper einer Vielfalt von Variationen unterliegen, bedingt abhängig von der aufgenommenen Menge ggf. eine schädigende Wirkung eines Fremdstoffes auf den Organismus.

Am 14.10.2021 hat die Europäische Kommission die EU-Chemikalienstrategie für Nachhaltigkeit im Rahmen des „Green Deal“ verabschiedet. Sie verfolgt das „Null-Schadstoff-Ziel“ für eine schadstofffreie Umwelt. Dies hört sich großartig an. Gleichzeitig ruft eine solche Formulierung bei Toxikologinnen und Toxikologen zunächst ein großes Fragezeichen hervor, denn ein jeglicher Stoff kann zum Schadstoff werden, wenn die Dosis nur hoch genug ist. Außerdem gibt es auch giftige Stoffe natürlichen Ursprungs, wie z. B. Schwermetalle und pflanzliche oder tierische Toxine, die bereits ohne menschliches Zutun in der Umwelt vorkommen und aufgenommen werden. Wahrscheinlich handelt es sich bei der Übersetzung der im Englischen verwendeten Formulierung „toxic-free environment“ als „schadstofffreie Umwelt“ also um eine etwas unscharfe Formulierung oder Übersetzung. Sie könnte so interpretiert werden, dass es ein Ziel sein muss, bezogen auf unsere natürlichen Ressourcen nachhaltiger zu handeln und dafür zu sorgen, dass wir die Umwelt nur mit solchen Stoffen bzw. Stoffmengen belasten, die unserer Gesundheit und Umwelt auch langfristig nicht schaden.

Europa hat hier bereits wichtige Weichen gestellt und mittlerweile für die meisten Bereiche (Lebensmittel, Trinkwasser, Chemikalien, Pflanzenschutzmittel, Biozide, Kosmetika, Verbraucherprodukte, Bauprodukte, Arzneimittel, Medizinprodukte etc.) stoffrechtliche Regelungen in Form von EU-weit gültigen Verordnungen etabliert. Grundsätzlich soll damit rechtlich sichergestellt werden, dass Mensch und Umwelt durch in Verkehr gebrachte chemische Stoffe und Erzeugnisse nicht geschädigt werden.

Aber es bestehen noch große Wissenslücken, so z. B. in Bezug auf:

- die Bewertung der gleichzeitigen Aufnahme von Fremdstoffen aus unterschiedlichen Quellen und über unterschiedliche Aufnahmepfade,
- die Entstehung und Freisetzung der nicht durch die Zulassungsverfahren erfassten Umweltkontaminanten oder
- die Mischungstoxizität, also die sich möglicherweise gegenseitig verstärkenden Effekte verschiedener Chemikalien mit ähnlicher Wirkung.

Wichtige staatliche Aufgaben im Sinne der Daseinsvorsorge und präventiven Gefährdungsminimierung sind es, zu prüfen, ob die schon bestehenden Regelungen bereits ihr Ziel erreichen, mögliche Lücken im Bewertungssystem aufzuzeigen und erforderliche Maßnahmen zum Schutz der Bevölkerung zu ergreifen. Diese Aufgaben erfordern jedoch die Kenntnis darüber, welche Stoffe aufgenommen werden, in welchen Konzentrationen diese im Körper vorkommen und ob sich dadurch ein Risiko für die menschliche Gesundheit entwickeln kann.

Eine zentrale Aufgabe für die systematische und wissenschaftliche Erhebung der aktuellen Belastung der Bevölkerung und der gesundheitlichen Bewertung dieser Daten hat in Deutschland das Umweltbundesamt. Im Rahmen regelmäßiger bevölkerungsrepräsentativer Studien (German Environmental Surveys – GerES) führt es neben Untersuchungen der Fremdstoffgehalte in der Innenraumluft und im Trinkwasser insbesondere Human-Biomonitoring-Untersuchungen durch.

Mithilfe des *Human-Biomonitorings (HBM)* ist es möglich, Fremdstoffe und ihre Abbauprodukte (Metaboliten) in Körpermedien, meist Urin, Blut oder Muttermilch, nachzuweisen und die Höhe der körperlichen Belastung zu bestimmen. Damit unterscheidet es sich grundsätzlich vom Umweltmonitoring, das Stoffe in Umweltmedien, wie z. B. der Luft, dem Trinkwasser, Lebensmitteln oder Kosmetika, erfasst, jedoch noch keine Aussage über die tatsächliche Aufnahme durch den Menschen zulässt und in der Regel auch nur den jeweiligen isolierten Expositionspfad betrachtet und so mögliche weitere Expositionsquellen unberücksichtigt lässt. Human-Biomonitoring untersucht die integrierte Belastung der untersuchten Person oder Bevölkerungsgruppe.

Die Erhebungen des Umweltbundesamtes werden seit Anfang der 1980er-Jahre durchgeführt. In 5 GerES-Studien wurden mittlerweile Urinproben von insgesamt ca. 16.700 Menschen auf eine Vielzahl von Umweltstoffen, wie z. B. die Schwermetalle Blei, Cadmium und Quecksilber, persistente organische Verbindungen (POPs) wie Dichlordiphenyltrichlorethan (DDT) und Polychlorierte Biphenyle (PCB) oder auch Weichmacher für Kunststoff, untersucht. GerES VI wird voraussichtlich in Kürze beginnen.

Mithilfe der Umweltprobenbank des Bundes können auch rückwirkend und über die zeitliche Entwicklung Aussagen zur Belastung des menschlichen Körpers mit Fremdstoffen getroffen werden. Sie dient daher auch als ergänzendes Instrument für die Erfolgskontrolle bestehender Regulierungen. Die Umweltprobenbank wurde ebenfalls zu Beginn der 1980er-Jahre etabliert. Sie ist die älteste Probenbank weltweit, die sowohl menschliche als auch Umweltproben ausschließlich mit dem Ziel sammelt und lagert, Umweltbelastungen zu untersuchen, und umfasst mehr als 280.000 Urin‑, Blut- und Plasmaproben der letzten 40 Jahre.

Bevölkerungsbezogene HBM-Daten werden aber auch von den Ländern oder auf kommunaler Ebene erhoben, z. B. in Muttermilchuntersuchungsprogrammen oder bei lokalen Ereignissen, um mögliche Belastungen betroffener Bevölkerungsgruppen zu erkennen und ggf. Maßnahmen zu ergreifen, sowie im Rahmen klinisch-umweltmedizinischer Untersuchungen von Einzelpersonen.

Sobald Untersuchungsmethoden etabliert wurden, können entsprechende Fremdstoffe und ihre Metaboliten im menschlichen Körper gemessen werden. Um jedoch ein mögliches gesundheitliches Risiko feststellen zu können, werden Informationen zur Giftigkeit (Toxizität) der Stoffe benötigt. Vor fast 30 Jahren wurde deshalb die *Human-Biomonitoring-Kommission (HBM-K)* ins Leben gerufen, die heute das Umweltbundesamt in allen Fragen des Human-Biomonitorings berät und mit der Aufgabe betraut ist, Konzepte und ein abgestimmtes wissenschaftliches Vorgehen für die toxikologische Bewertung von im Körper gemessenen Substanzen zu entwickeln. Die HBM‑K berät das Umweltbundesamt weiterhin bei der Anwendung der festgelegten Standards.

Die HBM-Kommission ist ein interdisziplinäres Gremium aus aktuell 13 unabhängigen Wissenschaftlerinnen und Wissenschaftlern aus Forschungseinrichtungen und Behörden sowie Gästen aus dem Umweltbundesamt (UBA), dem Bundesinstitut für Risikobewertung (BfR), dem Robert Koch-Institut (RKI), der Länderarbeitsgruppe Umweltbezogener Gesundheitsschutz (LAUG) sowie dem Bundesministerium für Umwelt, Naturschutz, nukleare Sicherheit und Verbraucherschutz (BMUV) und dem Bundesgesundheitsministerium (BMG). Die Mitglieder werden für jeweils 3 Jahre vom Umweltbundesamt berufen, zuletzt für die Periode 2020–2023. Die Geschäftsführung liegt in der Abteilung Umwelthygiene und ist dem Fachgebiet „Toxikologie, gesundheitsbezogene Umweltbeobachtung“ zugeordnet.

Statistische Referenzwerte und toxikologisch begründete Human-Biomonitoring-Werte (HBM-Werte) sind die Kernkonzepte der HBM-Kommission und die Basis zur Einordnung und gesundheitlichen Bewertung von bevölkerungsbezogenen HBM-Daten. Die zugrunde liegenden Konzepte werden regelmäßig überprüft, überarbeitet und erweitert, auch um aktuelle methodische Weiterentwicklungen in der regulatorischen Toxikologie aufzugreifen.

Einen Einblick in die Arbeitsbereiche der HBM-Kommission möchten wir Ihnen in dieser Ausgabe des Bundesgesundheitsblattes geben. Dazu finden Sie 3 Artikel unter der Rubrik „Bekanntmachungen – Amtliche Mitteilungen“ und einen unter „Forschung aktuell“.

Beispiele für das Vorgehen zur Ableitung der toxikologisch begründeten vorsorgeorientierten HBM-I-Werte für die Pyrethroidpestizide Deltamethrin und Cyfluthrin sowie das als Antioxidans und Lebensmittelzusatzstoff verwendete Butylhydroxytoluol finden Sie in den Artikeln „Ableitung von HBM-I-Werten für Deltamethrin und Cyfluthrin“ und „Ableitung eines HBM-I-Wertes für Butylhydroxytoluol (BHT) für Erwachsene“.

Die Ableitung von HBM-Werten für krebserzeugende Stoffe ohne einen Schwellenwert, unterhalb dessen eine sichere Konzentration anzunehmen ist, sieht das HBM-Wert-Konzept nicht vor. Um dennoch auch Funde dieser besonders besorgniserregenden Stoffe im Human-Biomonitoring bewerten zu können, hat die HBM-Kommission in Ergänzung zu den HBM-Werten ein erweitertes Konzept zur Bewertung karzinogener Stoffe entwickelt, das in dieser Ausgabe vorgestellt wird („Konzept für die Bewertung von krebserzeugenden Stoffen im bevölkerungsbezogenen Human-Biomonitoring“).

Aktuell überarbeitet die HBM-Kommission auch die Methodik ihrer Referenzwertableitung. Der mathematische Algorithmus, der der Festlegung der Referenzwerte zugrunde liegt, wird dafür genauer definiert, um die Ableitung transparenter und nachvollziehbarer zu machen. Das überarbeitete Konzept wird in einer der nächsten Ausgaben des Bundesgesundheitsblattes erscheinen.

Das Konzept des Human-Biomonitorings als wichtiges Instrument der gesundheitsbezogenen Umweltbeobachtung ist mittlerweile auch auf europäischer Ebene verankert. Dies ist insbesondere deshalb bedeutsam, weil die Zuständigkeit für die Chemikalienregulierung auf der EU-Ebene liegt und für ganz Europa geltende Richtlinien erlassen und Zulassungen etwa für Pflanzenschutzmittel- oder Biozidwirkstoffe erteilt werden. Das Umweltbundesamt hat hier in den letzten 5,5 Jahren die Europäische Human-Biomonitoring Initiative HBM4EU, ein gefördertes EU-Projekt mit einem Finanzvolumen von 74 Mio. € unter „Horizon 2020“, koordiniert. HBM4EU hat ein ambitioniertes Forschungsprogramm rund um offene, politikberatungsrelevante Fragen entwickelt, die seit der Projektvorbereitungsphase von EU-Institutionen und den 30 beteiligten Ländern identifiziert worden sind. Diese beziehen sich auf 18 bedeutsame Stoffe und Stoffgruppen, die für die Regulation und das Chemikalienmanagement besonders bedeutsam sind.

HBM4EU hat erstmalig für ganz Europa und Israel umfassende, auf höchstem Niveau qualitätsgesicherte Belastungsdaten für prioritäre Stoffe erhoben. Alle für qualitätsgesicherte HBM-Studien notwendigen Schritte wurden vor der Implementierung bearbeitet und harmonisiert. Die nun für Europa vorliegenden HBM4EU-Ergebnisse zeigen, dass trotz der elaborierten Chemikalienpolitik in der EU noch einiges zu tun ist, um die Menschen in Europa sicher vor gesundheitlichen Beeinträchtigungen durch Umweltchemikalien zu schützen. HBM4EU zeigt weiterhin, dass die Unterschiede in Nord‑, Ost‑, Süd- und West-Europa so groß sind, dass für die europäische Regulierung auch ein dauerhaft implementiertes europaweites HBM-System notwendig ist.

HBM4EU hat mit einer harmonisierten Methodik eine erste Datengrundlage geschaffen, die es ermöglicht, die Erfolge (oder unzureichenden Erfolge) bei der Umsetzung der Chemikalienstrategie zur Nachhaltigkeit zu messen. Die HBM-Kommission hatte im Rahmen von HBM4EU als Teil des National Hubs u. a. die Aufgabe der Beratung der nationalen Kontaktstelle (National Hub Contact Point) im Umweltbundesamt.

Das EU-Projekt war erfolgreich: Auch in Ländern, die HBM-Studien bereits durchgeführt haben, aber keine nationale Koordinierung der HBM-Aktivitäten hatten, ist nun ein für erfolgreiche und effiziente Arbeit notwendiges Netzwerk an der Schnittstelle zwischen Wissenschaft und Politikberatung (national und auf EU-Ebene) etabliert worden. Eine kurze Rückschau auf dieses wichtige Projekt bezüglich seiner Bedeutung für die Implementierung von Human-Biomonitoringstudien und in Hinblick auf das Erreichen der europäischen Nachhaltigkeitsziele im Rahmen des „Green Deal“ gibt der Artikel „Human-Biomonitoring für Europa“.

HBM4EU wird in dem noch größeren Projekt „European Partnership for the Assessment of Risks from Chemicals“ (PARC) fortgeführt. Dieses beinhaltet ein umfangreiches toxikologisches Forschungsprogramm, das neben der Forschung zu Humanaspekten auch die Umwelt einbezieht. PARC hat am 01.05.2022 begonnen und wird von der französischen Behörde für Lebensmittelsicherheit, umweltbezogenen Gesundheitsschutz und Arbeitsschutz (ANSES) koordiniert. Auch im Rahmen von PARC wird das Umweltbundesamt das Thema HBM auf europäischer Ebene voranbringen.

Die Belastungsdaten nützen nicht nur der Chemikalienregulierung, sondern auch der Information von Fachleuten und der allgemeinen Bevölkerung. Ohne bevölkerungsrepräsentative Referenzwerte lässt sich nicht beurteilen, ob in einem individuellen Fall oder an einem vermeintlichen Hotspot tatsächlich überdurchschnittliche Belastungen vorliegen. Diese Information ist handlungsleitend für nationale Behörden, aber auch für individualmedizinische Einschätzungen.

Für die Bevölkerung liefern HBM-Studien nicht nur den Mehrwert einer besseren Chemikalienpolitik, vielmehr geben die Ergebnisse auch vielfältige Hinweise, was Menschen tun können, wenn sie Belastungen mit Chemikalien möglichst vermeiden oder reduzieren möchten. Und vor allem schaffen HBM-Studien, deren Befunde toxikologisch gut bewertet sind, Transparenz in Bezug auf Belastungen, vermutete und wissenschaftlich begründete Risiken und die tatsächlichen Erfolge der Chemikalienpolitik.

Wir wünschen Ihnen eine interessante Lektüre.

Ihre

Claudia Röhl (Vorsitzende der HBM-Kommission) und Marike Kolossa-Gehring (Fachgebietsleiterin „Toxikologie, gesundheitsbezogene Umweltbeobachtung“)

